# Plasticity in centromere organization and kinetochore composition: Lessons from diversity

**DOI:** 10.1016/j.ceb.2021.12.007

**Published:** 2022-02

**Authors:** Midori Ishii, Bungo Akiyoshi

**Affiliations:** Department of Biochemistry, University of Oxford, UK

## Abstract

Kinetochores are the macromolecular protein complexes that govern chromosome movement by binding spindle microtubules during mitosis and meiosis. Centromeres are the specific chromosomal regions that serve as the platform on which kinetochores assemble. Despite their essentiality for proper chromosome segregation, the size and organization of centromeres vary dramatically between species, while different compositions of kinetochores are found among eukaryotes. Here we discuss recent progress in understanding centromeres and kinetochores in non-traditional model eukaryotes. We specifically focus on select lineages (holocentric insects, early diverging fungi, and kinetoplastids) that lack CENP-A, a centromere-specific histone H3 variant that is critical for kinetochore specification and assembly in many eukaryotes. We also highlight some organisms that might have hitherto unknown types of kinetochore proteins.

## Introduction

**Figure 1:**
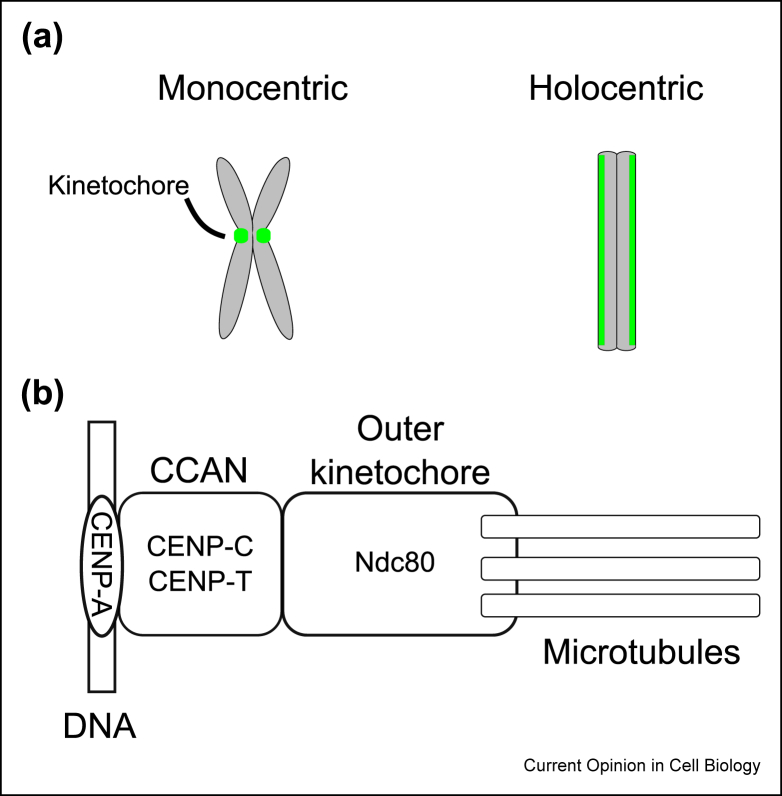
Monocentric and holocentric chromosomes. (**a**) In monocentric chromosomes, the centromere activity is confined to one region (left). In holocentric chromosomes, the centromere activity is scattered across the length of chromosomes (right). (**b**) A highly simplified schematic of a canonical kinetochore. Kinetochore position is typically determined by CENP-A-containing nucleosomes, upon which CCAN components assemble. CCAN recruits outer kinetochore components including Ndc80 complexes that bind microtubules.

Centromeres were first recognized cytologically as primary constrictions on each chromosome [1]. They represent the regions of the chromosomal DNA on which macromolecular kinetochore complexes assemble. Kinetochores govern chromosome segregation by mediating the interaction with spindle microtubules during mitosis and meiosis (Figure 1a) [2,3]. In many eukaryotes studied thus far, centromeres are determined epigenetically by the presence of CENP-A (also known as CenH3), a centromere-specific histone H3 variant, which also plays an important role in recruiting other kinetochore proteins onto centromeres [4] (Figure 1b). The Ndc80 complex forms a primary contact point for microtubules [5]. Bioinformatic searches identified putative homologs of these kinetochore proteins in diverse eukaryotes, suggesting that most eukaryotes use CENP-A to specify kinetochore positions and Ndc80 complexes to mediate microtubule attachment [6, 7, 8, 9].

**Figure 2:**
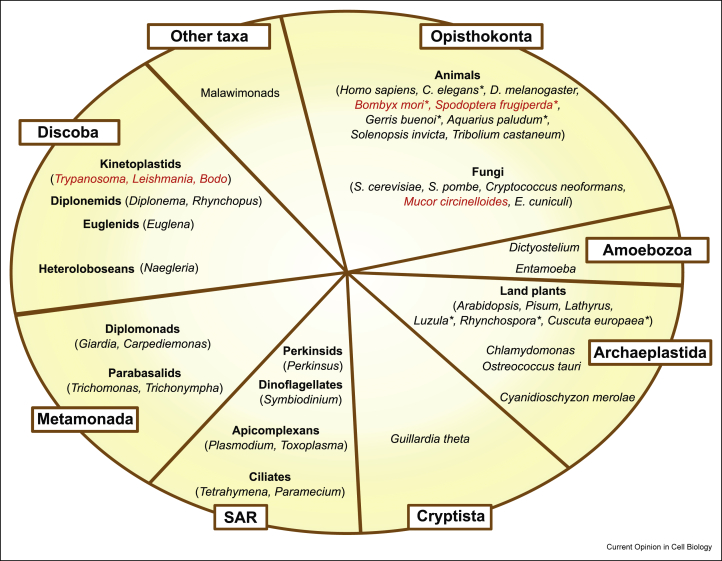
Current classification of eukaryotes. The classification is based on [10]. Select representative organisms, genera, or group names are shown as examples. Species that lack CENP-A are highlighted in red. Asterisks indicate holocentric species. SAR stands for stramenopiles, alveolates, and rhizarians.

Kinetochore research has mainly been carried out in select model eukaryotes such as yeasts, worms, flies, frogs, and humans. However, these organisms all belong to the supergroup Opisthokonta, meaning that these well studied fungi and animals do not necessarily represent the actual diversity of eukaryotes (Figure 2) [10]. In fact, identification of kinetochore components in deeply diverged eukaryotes started revealing radically different pictures of kinetochores. Furthermore, studies of less traditional model fungi and animals also highlighted the plasticity of centromeres and kinetochores [11]. In this review, we discuss latest findings from diverse eukaryotes.

## Diversity in centromere organization

Despite its essentiality for genetic inheritance, the DNA sequence at centromeres evolve rapidly [12,13]. Centromeres can be classified into two main types: monocentric and holocentric (Figure 1a) [14,15]. In monocentric chromosomes, kinetochore formation is confined to one region, while holocentric chromosomes assemble kinetochores along their length [16]. Although it remains unknown what kind of centromeres was present in the last eukaryotic common ancestor, it is generally thought that holocentricity is a derived feature [15,17].

**Figure 3:**
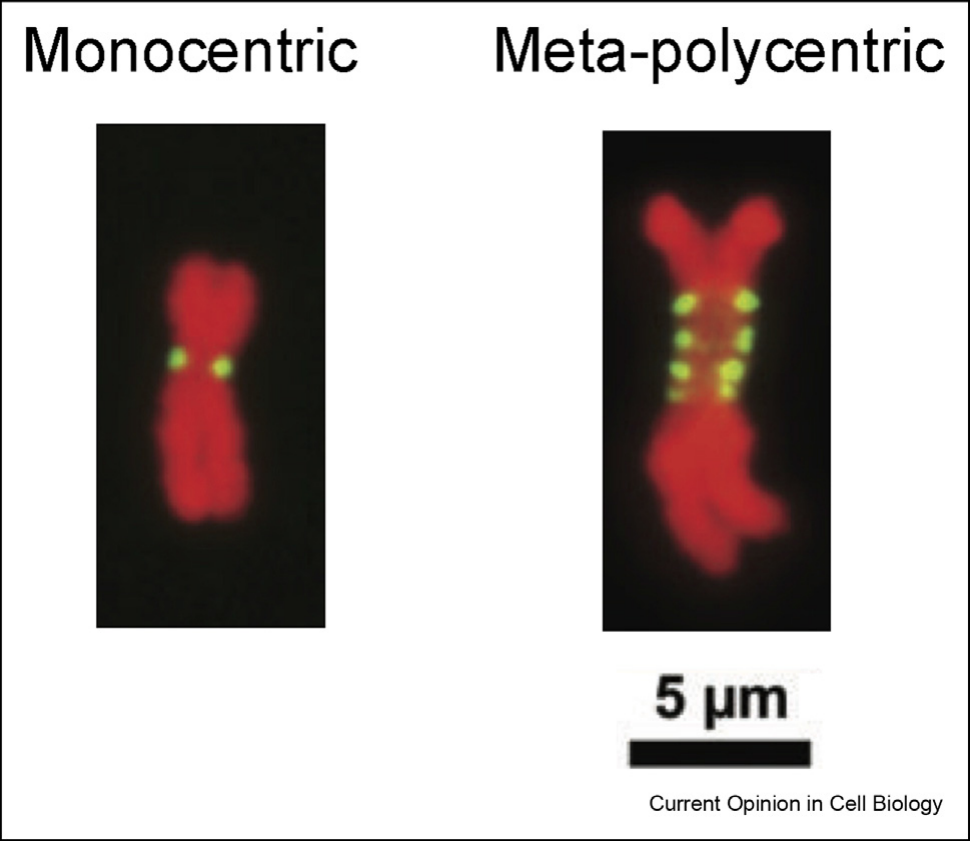
Meta-polycentric chromosomes have multiple cytologically distinct CENP-A domains. Examples of plant species that is monocentric (*Vicia sativa*, left) and meta-polycentric (*Lathyrus sativus*, right) are shown. Red: chromosome, green: CENP-A (CenH3). Adapted from Ref. [21] under CC-BY-NC 4.0 license.

In monocentric species, it is critical that only a single centromere is present per chromosome because dicentric chromosomes that have two functional centromeres are usually mitotically unstable [18]. The size of monocentromeres varies drastically between species. For example, the budding yeast *Saccharomyces cerevisiae* has a 125-bp point centromere [19], while humans have several Mbp regional centromeres [20]. The position of active centromeres in most species is marked by the presence of CENP-A whose immunostaining typically shows a single domain (Figure 3, left). However, multiple cytologically distinct CENP-A domains, called meta-polycentric (Figure 3, right), are found in certain plants (*Pisum sativum* and *Lathyrus* [21,22]), the red imported fire ants (*Solenopsis invicta* [23]), and the red flour beetle (*Tribolium castaneum* [24]) (see Figure 2 for the classification of each organism mentioned in this review). Meta-polycentromeres have extended primary constrictions and can occupy as much as 50% of the length of chromosomes [23], which might represent an intermediate transition state to holocentricity. The presence of only one primary constriction per chromosome implies that multiple cytologically distinct CENP-A domains end up forming one functional kinetochore unit. This is conceivably similar to dicentric chromosomes that can be stably maintained when the two centromeres are physically close to each other (e.g. up to ∼20 Mbp in human [25] and ∼1 kb in budding yeast [26]).

**Figure 4:**
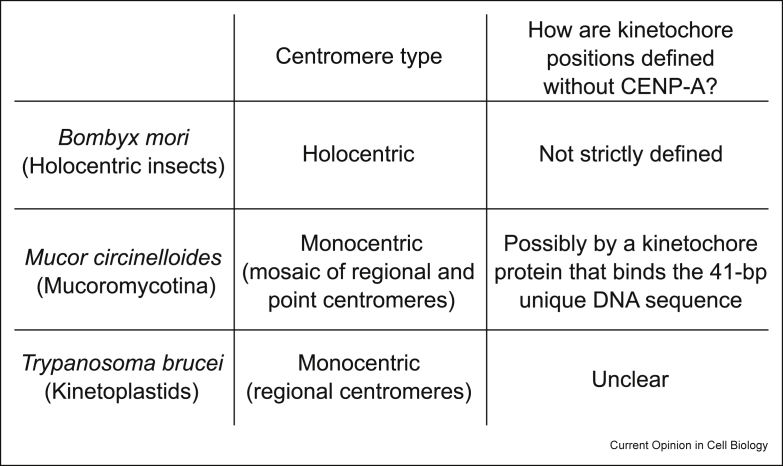
List of known organisms that lack CENP-A. See text for details.

In holocentric organisms, kinetochores assemble along the extensive segments of chromosomes, and spindle microtubules attach almost the entire surface of chromosomes [27]. It is likely that holocentric organisms, unlike monocentric ones, do not have the need to strictly specify kinetochore positions. However, CENP-A is present in most holocentric species including *C. elegans* [28] and holocentric plants [29], suggesting that CENP-A is important for kinetochore assembly in not only monocentric but also holocentric organisms. Interestingly, CENP-A is restricted to only one to three regions per chromosome in the holocentric plant *Cuscuta europaea*, which implies the presence of a CENP-A-independent mechanism to assemble kinetochores in this organism [30]. Furthermore, CENP-A is absent in certain lineages including holocentric insects, early diverging fungi, and kinetoplastids (Figure 2, Figure 4), meaning that these organisms assemble kinetochores and/or specify kinetochore positions without CENP-A.

## Most holocentric insects lost CENP-A

There are at least four insect lineages that transitioned from monocentric to holocentric states [17]. Detailed genomic and transcriptomic analyses of various insect lineages revealed that nearly all holocentric insects lack CENP-A and its binding partner CENP-C, while monocentric insects retain them [31], suggesting that these eukaryotes can assemble kinetochores without CENP-A and CENP-C. Building on these findings, recent proteomic analyses in the holocentric Lepidoptera (silkworm *Bombyx mori* and fall armyworm *Spodoptera frugiperda*) identified outer kinetochore proteins as well as some CCAN (Constitutive Centromere-Associated Network) components including a highly divergent CENP-T-like protein [32] (Figure 1b). Knockdown analysis showed that kinetochore assembly depends on CCAN in *B. mori*. Therefore, these holocentric insects that lack CENP-A and CENP-C assemble kinetochores using CCAN components. It will be interesting to reveal which CCAN proteins directly bind DNA and how outer kinetochore proteins are recruited to CCAN in Lepidoptera.

The fact that these holocentric insects do not have CENP-A raises an interesting question about how holocentromeres are organized. A recent study addressed this question using genome-wide profiling of kinetochore proteins in *B. mori*, which revealed broad regions of occupancy that overlapped with regions of low chromatin turnover [33]. It is noteworthy that a similar distribution pattern is found in *C. elegans*, a holocentric organism that has CENP-A [34,35]. These observations suggest that, in these holocentric organisms, kinetochores can assemble non-specifically in the genome where nucleosome turnover is low regardless of the presence or absence of CENP-A. A corollary is that kinetochore assembly sites are defined only loosely in holocentric species, which could make CENP-A dispensable. However, CENP-A is retained in most holocentric species (e.g. *C. elegans* and holocentric plants) [17]. It is currently unclear why only holocentric insects lost CENP-A. Interestingly, based on accelerated rates of CENP-T protein sequence evolution observed even in monocentric insects, it has been speculated that ancestral insects might have had a higher potential for CENP-A-independent kinetochore formation [32]. Understanding the function of CENP-T in monocentric and holocentric insects could provide hints into this question. Furthermore, some holocentric Hemipteran insects (*Gerris buenoi* and *Aquarius paludum*) have putative CENP-A homologs [32], which might represent an evolutionary intermediate state (i.e. switched to a holocentric state but yet to lose CENP-A). Characterization of CENP-A in these holocentric insects could provide insights into the evolution of holocentricity and evolution of CENP-A-independent kinetochore assembly in eukaryotes.

## Mosaic centromere in an early diverging fungus that lacks CENP-A

With the advent of next-generation sequencing techniques, more than 2000 representative fungal genomes are available and the identity of centromeres has been predicted in over 60 fungal species, representing a rich source for comparative analysis [36]. A previous bioinformatics survey of kinetochore proteins in diverse eukaryotes discovered the putative absence of CENP-A in early diverging fungi that belong to the order Mucorales of the subphylum Mucoromycotina [8]. Mucoromycotina consists of three orders: Mucorales, Umbelopsidales, and Endogonalean. A recent study examined the genome of 55 Mucoromycotina species and found that CENP-A is absent in all Mucorales and Umbelopsidales, but is present in Endogonalean and other fungal clades [37]. This suggests that CENP-A was present in the last Mucoromycotina common ancestor but was lost in the common ancestor of Mucorales and Umbelopsidales. Genome-wide profiling of kinetochore proteins identified the position of kinetochore assembly sites in *Mucor circinelloides*, a genetically tractable pathogenic fungus that causes a serious infectious disease called mucormycosis. The identified centromeres consist of ∼1 kb core centromere surrounded by gene-free pericentric regions where transposable elements are present. Interestingly, a 41-bp unique DNA sequence motif, reminiscent of budding yeast point centromeres, was found in the core centromere of all chromosomes, suggesting that the centromere in *M. circinelloides* is a mosaic of point and regional centromeres [37]. We speculate that there is a kinetochore protein that recognizes the unique sequence to specify kinetochore positions in *M. circinelloides* and that invention of such a mechanism could have driven the loss of CENP-A in the common ancestor of Mucorales and Umbelopsidales. Identification of centromere sequences in other Mucoromycotina could provide an insight into why CENP-A is missing in Mucorales and Umbelopsidales, but not in Endogonalean.

## Unconventional kinetochores in kinetoplastids

Another known lineage that lacks CENP-A is kinetoplastids, a group of unicellular flagellated eukaryotes that include parasitic (e.g. *Trypanosoma brucei,* the causative agent of human African trypanosomiasis) and free-living species [38]. Although genomes of more than 50 kinetoplastids have been sequenced, bioinformatics analysis failed to find any canonical kinetochore protein, including CENP-A and Ndc80 [8,39, 40, 41]. Through a localization-based screen and immunoprecipitation coupled with mass spectrometry, a number of kinetochore proteins have been identified in *T. brucei*, namely KKT1–25 [42, 43, 44] and KKIP1–12 [45,46]. These proteins have no clear similarity to canonical kinetochore components and are not found outside kinetoplastids, suggesting that the ancestor of kinetoplastids invented a unique set of kinetochore proteins [47]. This possibility is in line with the distinct geometry of their sister kinetochores observed by electron microscopy: unlike non-kinetoplastid species that have ∼1 μm distance between sister kinetochores in metaphase (the space is called the inner centromere) [48], there is no such space in kinetoplastids [49,50]. Interestingly, KKT16–18 are divergent homologs of SYCP2 and SYCP3, which are axial element components of the synaptonemal complex (SC) [51]. The SC is a meiosis-specific zipper-like structure that assembles between homologous chromosomes to promote recombination. It has been proposed that the ancestor of kinetoplastids repurposed the meiotic machinery to assemble unique kinetochores [51]. It remains to be determined to what extent kinetoplastid kinetochores share functional and structural similarities with synaptonemal complexes.

*T. brucei* has regional centromeres that consist of AT-rich repetitive sequences (20–120 kb in size) [52]. Unlike *M. circinelloides*, there is no specific DNA sequence that is common to all centromeres in *T. brucei* or other kinetoplastids [53,54]. It is therefore thought that kinetoplastids determine kinetochore positions in a sequence-independent manner. However, essentially nothing is known about the nature of centromeric chromatin in kinetoplastids [55], and it remains a mystery how their kinetochores assemble specifically at centromeres. In other species, constitutive kinetochore components such as CENP-A and CCAN play crucial roles in kinetochore specification (Figure 1b). By analogy, constitutive components may carry out these functions in kinetoplastids. *T. brucei* has six constitutive kinetochore proteins (KKT2, KKT3, KKT4, KKT20, KKT22, and KKT23). KKT2 and KKT3 are homologous to each other and likely evolved from a polo-like kinase [43]. Besides an N-terminal protein kinase domain and C-terminal divergent polo boxes, KKT2 and KKT3 additionally have a central domain that is important for their centromere localization [56]. Recent crystal structures of the KKT2 central domain revealed that it is a unique structure. Despite these progresses, it remains unknown what kind of centromere-specific features are recognized by the KKT2/3 central domain. We speculate that centromere-specific histone modifications (e.g. phosphorylation by KKT2/3 or acetylation by KKT23 that has a GCN5-Related N-Acetyltransferase domain) might mark the position of kinetochore assembly sites in kinetoplastids that lack CENP-A.

Kinetoplastids are one of few lineages that apparently lack the microtubule-binding Ndc80 complex. So far KKT4 is the only kinetoplastid kinetochore protein that is known to have microtubule-binding activity [57]. In other species, microtubule-binding kinetochore proteins typically localize at the outer region of kinetochores during mitosis (e.g. Ndc80 (Figure 1b)). However, KKT4 constitutively localizes at the inner part of the kinetochore and has DNA-binding activity, highlighting the uniqueness of kinetoplastid kinetochores. Recent structural characterizations of KKT4 showed that its microtubule-binding domain consists of a coiled coil and a positively charged disordered tail [58]. In addition, KKT4 has a BRCT domain, which is not found in any canonical kinetochore protein in other eukaryotes. Although some KKIP proteins localize at the outer region of kinetoplastid kinetochores, it remains unknown whether any of them binds microtubules [46].

Protein kinases regulate various kinetochore functions in eukaryotes, and there are at least four protein kinases at kinetoplastid kinetochores. KKT10 and KKT19 have a CLK-like kinase domain, while kinase domains of KKT2 and KKT3 are classified as unique among eukaryotic kinase subfamilies [59]. Recent studies show that KKT10 and KKT19 regulate the metaphase-to-anaphase progression [60,61], which represents an interesting finding because *T. brucei* apparently lacks a canonical spindle checkpoint system [62,63]. It is therefore likely that these kinases regulate the cell cycle progression in a unique manner. Although numerous phosphorylation sites have been identified in kinetoplastid kinetochore proteins by mass spectrometry, little is known about the relevance of these phosphorylation events [60,64]. Further studies are needed to understand the regulation of kinetochores and the cell cycle in kinetoplastids.

## Conclusions

A wide variety of centromeres found in eukaryotes suggest that cells can manage to perform accurate chromosome segregation using different geometries of centromeres. Compositions of kinetochore proteins can also drastically vary. Moreover, it is important to remember that although bioinformatics can detect conserved kinetochore proteins in sequenced organisms, this approach cannot identify lineage-specific kinetochore proteins that are absent in traditional model eukaryotes. For example, lineage-specific kinetochore proteins have been experimentally discovered in *Cryptococcus neoformans* [65] and apicomplexan parasites *Toxoplasma* and *Plasmodium* [66]. To obtain a comprehensive picture of kinetochore compositions in eukaryotes, it is important to identify kinetochore proteomes in various eukaryotes.

Understanding kinetochore compositions in deeply diverged eukaryotes could reveal radically different kinetochores, as those found in kinetoplastids (Discoba). It is noteworthy that very few kinetochore proteins have been detected in some lineages such as diplonemids (Discoba) and *Carpediemonas membranifera* (Metamonada) [41,67]. It will be interesting to find out what kind of kinetochore proteins are present in these species. Another notable example of kinetochores is found in some dinoflagellates and perkinsids (SAR) as well as parabasalids (Metamonada). Although they apparently have canonical kinetochore proteins, their kinetochores are embedded in the nuclear membrane [7]. Understanding how (and why) these organisms embed kinetochores within membranes and how they achieve accurate chromosome segregation could provide additional insights into the diversity of kinetochores. With advances in techniques to study centromeres and kinetochores [68, 69, 70], it is very likely that we will learn a lot of lessons by studying diverse organisms in the next few decades.

## Open Access

This research was funded in whole, or in part, by the 10.13039/100010269Wellcome Trust [grant 210622/Z/18/Z]. For the purpose of Open Access, the author has applied a CC BY public copyright license to any Author Accepted Manuscript version arising from this submission.

## Conflict of interest statement

Nothing declared.
